# Personal perspective on *Environmental Health and Preventive Medicine*

**DOI:** 10.1186/s12199-017-0647-2

**Published:** 2017-03-31

**Authors:** Hidekuni Inadera

**Affiliations:** Department of Public Health, Faculty of Medicine, University of Toyama, 2630 Sugitani, Toyama 930-0194 Japan

First of all, as Editor-in-Chief, I want to welcome our readers to a new era of *Environmental Health and Preventive Medicine* (EHPM), the official journal of the Japanese Society for Hygiene. Since 1996, EHPM has been a major journal in the field of environmental medicine and public health, disseminating the latest exciting research in the field of environmental health and preventive medicine. EHPM takes a comprehensive approach to disease prevention and environmental health, covering medical, biological, molecular, genetic, physical, psychosocial, chemical, and other environmental factors [1].

In 2016, EHPM reached a significant milestone. Following continuous efforts by the ex-Editors-in-Chief, Professors A. Koizumi and T. Otsuki, the journal gained its first impact factor (IF) rating of 1.214. Although IF is just one indicator of a journal’s ranking, we are aware that scientists are evaluated by the cumulative IF of their publications. I hope that EHPM’s IF will rise steadily with the publication of high-quality manuscripts from a wide range of scientific fields. This will mean that the number of submitted manuscripts will also rise steadily, and we will continue to evaluate them properly in a thorough and transparent review process: we understand how important critical peer review and publication ethics are. Fortunately, active researchers from all over the world serve as peer reviewers for EHPM. We will also endeavor to keep the submission/review/decision cycle as short as possible, but without sacrificing quality. Speedy publication will help ensure that EHPM papers are read and cited more often by experts in relevant fields. Also, we aim to help our readers discern valuable information in today’s world of ever-increasing information sources, by publishing high-impact review articles from experts in all fields related to environmental health and preventive medicine.

As part of our efforts to help bolster EHPM’s position as a keystone journal, in 2016 we established a best reviewer award to encourage participation in peer review [2]. Each year, the best reviewer will be selected from among those who provided the most quality reviews, as evaluated by the Editor-in-Chief, within the stated deadlines. Furthermore, to acknowledge authors who have produced quality articles, we will name the original article with the most downloads in the 12 months after its publication in EHPM at the annual meeting of the Japanese Society of Hygiene.

As a second major milestone, EHPM is to become an open access journal, beginning with the first issue of 2017. All accepted manuscripts will be published in BioMed Central as open access articles. In open access format, EHPM manuscripts will be read, and hopefully cited, by a much larger audience. We encourage you to submit your work: even a single study can prove invaluable if it triggers a major breakthrough in the field. And as long as that study has a sound scientific basis, it can serve to encourage further and related research. It is my hope that such high-impact studies will be published in EHPM.

Asia is the world’s most populous continent and home to the most rapidly industrializing nations in the world. Countries in Asia share many common characteristics, including historical background and natural environment. Nowadays, the adverse environmental health consequences of rapid industrialization and development have become obvious in many areas across the continent. Therefore, EHPM welcomes articles relevant to environmental health in Asia. Accumulating scientific evidence indicates that environmental risk factors, such as even very low levels of environmental exposure to chemical contaminants, can profoundly affect human health. In fact, the G7 Environment Ministers’ meeting, which was held in Toyama, Japan, in May 2016, emphasized the commitment to international cooperation for better understanding of chemical risks, especially with respect to children’s health and development. Accordingly, EHPM would also like to publish articles in the active research area of awareness raising and evaluation of the effects of environmental contaminants.

Environmental issues such as global climate change are universal, and multinational collaboration is not merely an option but a necessity. Moreover, the Fukushima Daiichi nuclear disaster has shown that an environmental disaster in one country can profoundly affect other countries. In this age of globalization, EHPM seeks to develop broader and deeper international exchange and collaboration. Appropriate solutions can be achieved by communication and collaboration among scientists and policy-makers facing similar issues. Because the population of Asia is increasing and air pollution is worsening, we hope that EHPM will be a catalyst for increased collaboration between Asian researchers and scientists on problems of Asian environmental health. Our efforts have begun with the internationalization of the Editorial Board, which has members from a number of Asian countries.

From a public health perspective, it is clear that smoking is currently the greatest behavioral risk factor for non-communicable diseases (NCDs). In light of concerns that sources of funding can influence study outcomes in invisible ways and that it is virtually impossible for peer review to detect bias and research misconduct, some journals no longer consider studies that are funded in whole or part by the tobacco industry [3, 4]. Whether EHPM will follow suit must be determined according to our own editorial ethics and will be discussed more deeply among the board members of EHPM.

Finally, I want to acknowledge the hard work of the editors. Their dedication to maintaining a fair and constructive peer review process enables us to publish high-quality, meaningful articles. Certainly, receiving an IF rating and becoming an open access journal are both important events for EHPM. Now is the time to put our heads together to help EHPM flourish in its commitment to serve the environmental science community.
